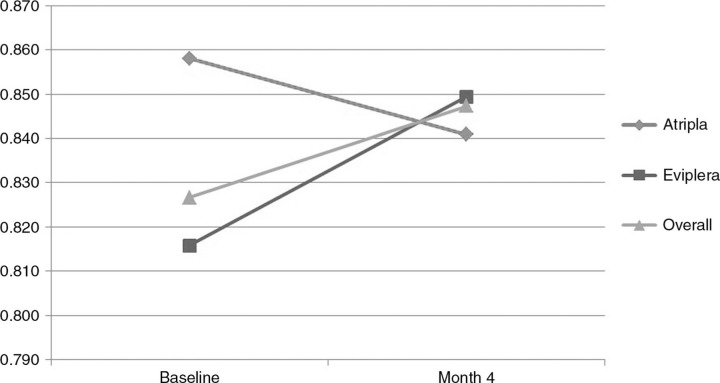# Switching from an EFV-based STR to a RPV-based STR is effective, safe and improves HIV patients health status

**DOI:** 10.7448/IAS.17.4.19798

**Published:** 2014-11-02

**Authors:** Franco Maggiolo, Sergio Di Matteo, Giacomo Bruno, Noemi Astuti, Elisa Di Filippo, Daniela Valenti, Giorgio Colombo

**Affiliations:** 1 Infectivology, Azienda Ospedaliera Papa Giovanni XXIII, Bergamo, Italy; 2 Studi Analisi Valutazioni Economiche (SAVE), Milano, Italy; 3 Drug Science, School of Pharmacy, Pavia, Italy

## Abstract

**Introduction:**

TDF/FTC/RPV has been shown effective in both naïve and PI-pre-treated patients. Less is known about a switch strategy in subjects receiving EFV.

**Materials and Methods:**

We evaluated viro-immunologic outcomes, Quality of Life (QoL) and costs of an unselected cohort of patients switching from a TDF/FTC/EFV STR (≥6 months duration) to a TDF/FTC/RPV STR. The considered outcome measures were quality-adjusted life years (QALYs) as measured with the EQ5D questionnaire and the overall direct health costs. 64 patients with a baseline viral load<50 copies/mL were randomized to immediately switch therapy or to continue TDF/FTC/EFV for four months and then switch to TDF/FTC/RPV. Six patients in the deferred switch group did not actually change cART.

**Results:**

Patients were mostly males (73.4%) with a mean age of 46 years, a baseline mean HIV-RNA of 6.4 copies/mL and a mean baseline CD4 count of 588 cells/µL. For the considered follow-up period, the mean cost per patient resulted 2,563 for TDF/FTC/RPV and 2,572 for TDF/FTC/EFV. Viremia remained undetectable and CD4 stable in all patients. Over time the mean QoL increased in the RPV arm ad slightly decreased in the EFV arm, after four months the mean per patient QALYs was 0.849 for RPV and 0.841 for EFV, respectively (Figure 1). A sharp increment of QoL was observed in the deferred-switch arm after switch, too. VAS analysis of health status perception also increased overall from 82.78 to 83.79 due to the improvement in the RPV arm. Mean cholesterol levels improved in the RPV arm from 203 to 170 mg/dL, while an increment from 190 to 207 mg/dL was observed in the EFV arm. HDL levels lowered from 49 to 45 and rose from 53 to 54 mg/dL in the RPV and EFV arms, respectively. Triglycerides levels improved both in the RPV arm (from 138 to 112 mg/dL) and in the EFV arm (from 110 to 103 mg/dL).

**Conclusions:**

Switching from TDF/FTC/EFV to TDF/FTC/RPV is a safe, well tolerated strategy that improves the overall health status of HIV-treated patients. The switch does not expose patients to a risk of virologic failure due to possible PK interactions of the drugs. RPV compared to EFV proved to be cost-effective showing lower cost and higher outcome measure values.

**Figure 1:**